# A Specific Remedy for Cholera Asiatica

**Published:** 1886-04

**Authors:** 


A SPECIFIC REMEDY FOR CHOLERA ASIATICA.

Regnoli, of Rome, has employed Aethiop's mineral (black sulphuret of mercury) in the recent epidemic of cholera in Italy. Of 241 patients treated, 223, or 95 per cent., were cured. In the preliminary stage of diarrhœa, thirty grains or less were given in one dose. If the disease is more advanced, even larger doses are given. *Il. Raccoglitore Med.*, Feb., 1886.

The following table shows the death rate per 1,000 from all causes and from zymotic diseases, in each of the wards in Buffalo, being an average of the mortality statistics for the five years, 1881 to 1885 inclusive:

| Wards. | All Causes. | Zymotic Diseases. | Percentage of Deaths due to Zymotic Diseases. |
|---|---|---|---|
| First, | 21½ | 7.1 | 35½ per ct. |
| Second, | 16½ | 3.8 | 24 " |
| Third, | 19 | 6.5 | 34 " |
| Fourth, | 17¾ | 5.2 | 29 " |
| Fifth, | 25 | 10.5 | 42 " |
| Sixth, | 22¾ | 8. | 33⅓ " |
| Seventh, | 19½ | 8.2 | 34 " |
| Eighth, | 22¾ | 6.2 | 26 " |
| Ninth, | 16½ | 3.2 | 19½ " |
| Tenth, | 12¼ | 5.7 | 27 " |
| Eleventh, | 16 | 5. | 28 " |
| Twelfth, | 20 | 9. | 24 " |
| Thirteenth, | 16 | 7.6 | 29 " |
| City, | 21⅗ | 6.95 | 32 " |

In estimating the death rate from all causes, the deaths in public institutions are deducted. In the death rate from zymotic diseases, the deaths in public institutions are included.

